# The curious case of IL‐33 in homeostasis and infection

**DOI:** 10.1002/eji.202049031

**Published:** 2020-12-17

**Authors:** Reza Nadafi, Ramon Arens

**Affiliations:** ^1^ Department of Immunology Leiden University Medical Center Leiden The Netherlands

**Keywords:** IL‐33, lymph node, stromal cells, CD8^+^ T cells, LCMV

## Abstract

The importance of interleukin (IL)‐33 in promoting effective antiviral immune responses is evident, yet the critical cellular sources of IL‐33 in homeostasis and infection are largely unknown. In this issue of the *European Journal of Immunology*, Aparicio‐Domingo et al. [Eur. J. Immunol. 2021. 51: 76–90] explore the main source of IL‐33 expression in lymph nodes (LNs) and dissect its role in LN homeostasis and antiviral adaptive immune response. The authors reveal that fibroblastic reticular cells and lymphatic endothelial cells are both producing IL‐33 in steady‐state LNs. Remarkably, however, by using cell‐type specific deletion approaches, the authors demonstrate that exclusively fibroblastic reticular cells, and not lymphatic endothelial cells, are the critical cellular source for promoting antiviral CD8^+^ T‐cell responses upon infection. These findings provide an important insight into the role of specific LN stromal cell subsets as potent modulators of antiviral immunity.

## Graphical abstract

Fibroblastic reticular cells (FRCs) are a subset of lymph node stromal cells that produce the alarmin IL‐33 upon lymphocytic choriomeningitis virus infection. The released IL‐33 **triggers**the IL‐33 receptor ST2 expressed by CD8^+^T cells, and this results in increased proliferation and production of IFN‐γ and TNF.

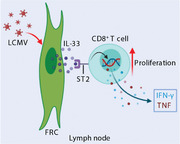

Interleukin (IL)‐33 has an intriguing dual function, as it acts as a proinflammatory extracellular cytokine while it can also regulate gene transcription as an intracellular nuclear factor [1, 2]. IL‐33, belonging structurally to the IL‐1 superfamily, is known as an “alarmin” that acts like other damage‐associated molecular pattern (DAMP) molecules upon tissue damage, infection, or necrosis [3]. IL‐33 is then rapidly released into the extracellular environment in an active form and targets cells expressing the IL‐33 receptor ST2 (IL1RL1) [4]. In steady‐state conditions however, IL‐33 is primarily prestored as a nuclear protein in nonhematopoietic cells, including endothelial cells and epithelial cells, but also in fibroblastic reticular cells of secondary lymphoid organs [3]. Several types of immune cells, including granulocytes, dendritic cells (DCs), macrophages, type 2 innate lymphoid cells (ILC2s), activated T cells and B cells, natural killer (NK) cells, NK T cells, and regulatory T cells (T_REGs_), feature ST2 expression [5]. Accordingly, signaling via the ST2 receptor leads to differentiation, maintenance, and effector function of several immune cells [4, 5, 6, 7]. In line with this, recent research has described a central role for IL‐33 in tumor immunity, metabolic disease, inflammation, and infection [4, 5, 6, 7, 8].

The central role of IL‐33 is underscored by affecting both innate and adaptive immune responses in various settings. For example, IL‐33 signaling through ST2 mediates the expression of cytokines such as IL‐4, IL‐5, and IL‐13, which are associated with type 2 immune responses by innate and adaptive immune cells [9, 10]. IL‐33 can also support T_REG_ development and their immunosuppressive function [11]. On the other hand, studies employing IL‐33 and ST2‐deficient mice have demonstrated that IL‐33 secretion drives protective antiviral CD8^+^ T‐cell responses and Th1 development in secondary lymphoid organs [12, 13]. Of note, nonhematopoietic cells in the splenic T‐cell zone were the source of IL‐33 production required for a potent antiviral CD8^+^ T‐cell response [12]. These data are consistent with previous studies highlighting the nonhematopoietic stromal cells in the T‐cell zone of spleen and lymph nodes (LNs) as main source of IL‐33 [3, 14]. However, the classical subdivision of nonhematopoietic stromal cells within the LN already reveals several distinct populations, including fibroblastic reticular cells (FRCs), follicular DCs (FDCs), marginal reticular cells (MRCs), lymphatic endothelial cells (LECs), blood endothelial cells (BECs) and the poorly characterized double negative cells (DNs) [15]. Hence, it is reasonable to speculate that one or more nonhematopoietic cell subset of the LN function as a vital source of IL‐33 impacting on antiviral CD8^+^ T‐cell responses. In this issue of the *European Journal of Immunology*, Aparicio‐Domingo et al. show that FRCs are the main source of IL‐33 that is required for an effective antiviral CD8^+^ T cell response (Fig. 1).

**Figure 1:**
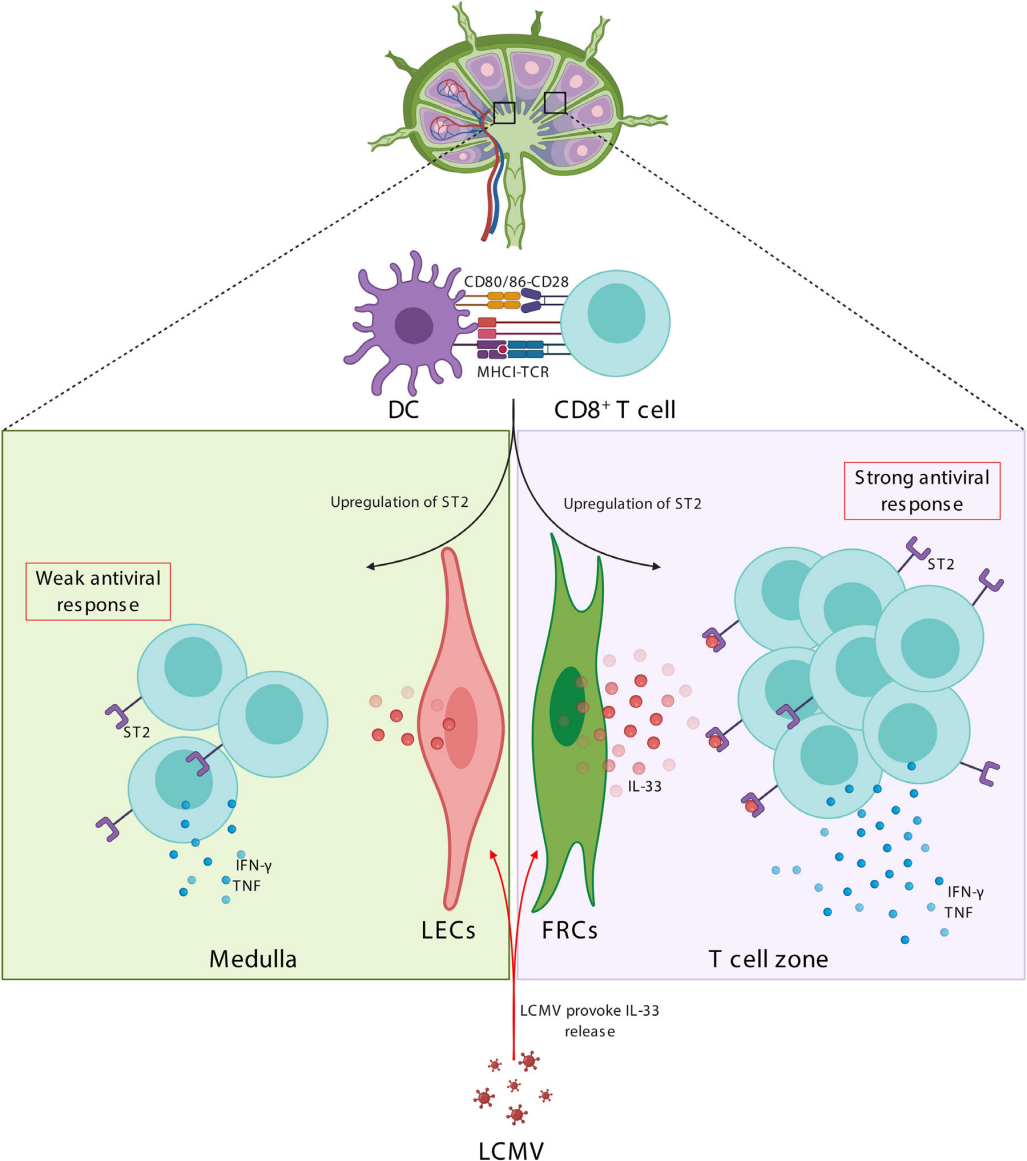
IL‐33 release by lymph node fibroblastic reticular cells promotes antiviral immune responses. Upon LCMV infection, antigen‐presenting cells such as dendritic cells (DCs) become activated and subsequently activate CD8^+^ T cells through presentation of LCMV antigens in the context of MHC‐I molecules and by providing costimulatory signals. The primed CD8^+^ T cells in lymph nodes (LNs) upregulate IL‐33 receptors (ST2) on their surface. Furthermore, lymph node stromal cells, in particular fibroblastic reticular cells (FRCs) in the T cell‐zone of the LN, release prestored IL‐33 into the extracellular matrix. The release of IL‐33 by FRCs but not lymphatic endothelial cells (LECs) results in promotion of effective antiviral CD8^+^ T cell responses. Figure was created with BioRender.com.

Next to providing structural support in the LN, stromal cells are also actively involved in migration and survival of lymphocytes, tolerance induction, and orchestrating adaptive immune responses during infections [15, 16]. It was previously shown that maturation of LN FRCs from myofibroblastic precursors is required for optimal support of adaptive antiviral immune responses [17]. Additionally, IL‐7 production by LN stromal cells, in particular FRCs, was reported essential for LN remodeling after systemic viral infection [18]. Considering the importance of the LN stromal cells and IL‐33 in antiviral immunity, Aparicio‐Domingo et al. provide further insights into the role of LN stromal cells in directing acute and chronic antiviral CD8^+^ T‐cell responses by identifying the specific stromal cell subset involved in IL‐33 production [19]. By using IL‐33‐GFP reporter mice, the authors first identified the prominent source of IL‐33 in the steady‐state LN. Histological staining for IL‐33 protein was mainly observed throughout the T cell‐zone and interfollicular region, which are inhabited by FRCs. In contrast, GFP expression was absent in B‐cell areas, suggesting that IL‐33 is not expressed by FDCs or other stromal cell subsets in B‐cell follicles. Additionally, IL‐33^+^ cells were also present in the medullary region of the LNs, mostly as PDPN^+^ and LYVE1^+^ expressing stromal cells, which represent FRCs and LECs, respectively. Indeed, flow cytometry and RT‐qPCR on stromal subsets from naïve mice confirmed T‐cell zone FRCs, medullary FRCs and LECs as the foremost sources of IL‐33 production. The expression of IL‐33 at the transcript and protein level is in line with previously published data [3, 14]. For example, in human spleen and LN stromal cells, expression of IL‐33 was mainly observed in the T‐cell zone, interfollicular region, and medullary stromal cells [3, 14]. Interestingly, Aparicio‐Domingo et al. report that cell number, organization, distribution, and gene expression of LN stromal cells, including FRCs, are similar in IL‐33‐deficient mice compared to wild‐type mice. This indicates that LN stromal cell development and maintenance occurs independently of IL‐33. Thus, despite being expressed by FRCs, IL‐33 does not appear to be involved in the homeostasis of these cells.

Whether IL‐33 comes into play in settings of disrupted homeostasis was investigated by Aparicio‐Domingo et al. in acute and chronic infection models with lymphocytic choriomeningitis virus (LCMV). In IL‐33^gfp/gfp^ (IL‐33 deficient) mice infected with LCMV clone 13, causing chronic infection, the authors observed a significant reduction in both frequencies and absolute numbers of LCMV‐specific effector CD8^+^ T cells compared to IL‐33^gfp/+^ mice, demonstrating that IL‐33 secretion by LN stromal cells promotes antiviral CD8^+^ T cell responses. This effect on CD8^+^ T‐cell responses coincided with a transient downregulation of IL‐33 transcripts and protein expression in LN stromal cells early after infection, which is likely a sign of IL‐33 secretion. To take a closer look at the stromal subset mediating the antiviral CD8^+^ T‐cell response, Aparicio‐Domingo et al. used tamoxifen‐inducible Cre recombinase systems to generate conditional knockout mice in which IL‐33 is deleted in either FRCs (*CcL19‐Cre*) or in LECs (*Prox1‐CreERT2*). Interestingly, the generation of antiviral CD8^+^ T cell responses was unaltered when IL‐33 was deleted specifically in LECs. However, FRC‐restricted deletion of IL‐33 resulted in significantly reduced IFN‐γ and TNF production by virus‐specific CD8^+^ T cells in LNs. Upon infection with LCMV WE strain causing acute infection, Aparicio‐Domingo et al. observed no changes in IL‐33 production in the stromal cells. Nonetheless, viral‐specific CD8^+^ T cells were significantly decreased in IL‐33‐deficient mice. Importantly, during acute LCMV infection, IL‐33 production by FRCs and not LECs was crucial to promote antiviral CD8^+^ T‐cell responses. Apparently, (transient) downregulation of IL‐33 in FRCs is not a requirement to affect CD8^+^ T‐cell responses. Together, these data underline that IL‐33 production by FRCs is essential for the generation of antiviral CD8^+^ T‐cell responses in both acute and chronic viral settings.

Based on the findings reported by Aparicio‐Domingo et al., it is tempting to conclude that LN stromal cells, in particular FRCs in the T‐cell zone, are the most prominent source of IL‐33 required for mounting CD8^+^ T‐cell responses upon viral infection. However, infection with LCMV is exceptional in certain aspects compared to other viral infections, for example, CD8^+^ T‐cell responses against LCMV are differentially dependent on costimulatory signals and type I interferons play a dominant role in driving LCMV‐specific CD8^+^ T‐cell responses [20]. Thus, whether the observations by Aparicio‐Domingo et al. can be translated to other viral infections, for example, vaccinia virus and MHV‐68 infections known to be controlled by IL‐33 [12], remains to be determined. Besides impacting on antigen‐specific CD8^+^ T cells, IL‐33 can also stimulate so‐called “ bystander” activated CD8^+^ T cells when combined with other cytokines such as IFN‐β, IL‐2, IL‐12, and IL‐15 [21, 22]. The in vivo role of possible bystander activating effects of IL‐33 are, however, currently unknown. Another intriguing aspect that would require further investigation is whether there is heterogeneity among FRC subsets with respect to detection of DAMPS and/or IL‐33 secreting capacity. Emerging single‐cell technology and computational methods enable the identification of cell diversity at single‐cell resolution. Such analyses already revealed phenotypic and functional heterogeneity of the T‐cell zone FRCs into three main subsets based on CCL19 and CCL21 expression [23]. Moreover, the mechanism and signaling pathways required for IL‐33 secretion by LN stromal cells requires further investigation. Certainly, the current report by Aparicio‐Domingo et al. has added a novel insight into the function of LN stromal cells in antiviral CD8^+^ T‐cell responses, and this may yield new directions for clinical applications. Targeting of IL‐33 or its cellular source may serve to be beneficial for empowering viral and tumor immunity [24, 25], given the importance of this intriguing cytokine for promoting CD8^+^ T‐cell expansion.

## Conflict of interest

The authors declare no financial or commercial conflict of interest.

**Abbreviations**
FRC: fibroblastic reticular cell
LCMV: lymphocytic choriomeningitis virus
LEC: lymphatic endothelial cell

## References

[1] Carriere, V. , Roussel, L. , Ortega, N. , Lacorre, D. A. , Americh, L. , Aguilar, L. , Bouche, G. and Girard, J. P. , IL‐33, the IL‐1‐like cytokine ligand for ST2 receptor, is a chromatin‐associated nuclear factor in vivo. Proc. Natl. Acad. Sci. USA 2007 104: 282–287. 17185418 10.1073/pnas.0606854104 PMC1765450

[2] Choi, Y. S. , Park, J. A. , Kim, J. , Rho, S. S. , Park, H. , Kim, Y. M. and Kwon, Y. G. , Nuclear IL‐33 is a transcriptional regulator of NF‐kappaB p65 and induces endothelial cell activation. Biochem. Biophys. Res. Commun. 2012 421: 305–311. 22708120 10.1016/j.bbrc.2012.04.005

[3] Pichery, M. , Mirey, E. , Mercier, P. , Lefrancais, E. , Dujardin, A. , Ortega, N. and Girard, J. P. , Endogenous IL‐33 is highly expressed in mouse epithelial barrier tissues, lymphoid organs, brain, embryos, and inflamed tissues: in situ analysis using a novel Il‐33‐LacZ gene trap reporter strain. J. Immunol. 2012 188: 3488–3495. 22371395 10.4049/jimmunol.1101977

[4] Cayrol, C. and Girard, J. P. , Interleukin‐33 (IL‐33): a nuclear cytokine from the IL‐1 family. Immunol. Rev. 2018 281: 154–168. 29247993 10.1111/imr.12619

[5] Peine, M. , Marek, R. M. and Lohning, M. , IL‐33 in T cell differentiation, function, and immune homeostasis. Trends Immunol. 2016 37: 321–333. 27055914 10.1016/j.it.2016.03.007

[6] Molofsky, A. B. , Savage, A. K. and Locksley, R. M. , Interleukin‐33 in tissue homeostasis, injury, and inflammation. Immunity 2015 42: 1005–1019. 26084021 10.1016/j.immuni.2015.06.006 PMC4471869

[7] Zhou, Z. , Yan, F. and Liu, O. , Interleukin (IL)‐33: an orchestrator of immunity from host defence to tissue homeostasis. Clin. Transl. Immunol. 2020 9: e1146. 10.1002/cti2.1146 PMC7299676 32566227

[8] Martin, N. T. and Martin, M. U. , Interleukin 33 is a guardian of barriers and a local alarmin. Nat. Immunol. 2016 17: 122–131. 26784265 10.1038/ni.3370

[9] Schmitz, J. , Owyang, A. , Oldham, E. , Song, Y. , Murphy, E. , McClanahan, T. K. , Zurawski, G. et al., IL‐33, an interleukin‐1‐like cytokine that signals via the IL‐1 receptor‐related protein ST2 and induces T helper type 2‐associated cytokines. Immunity 2005 23: 479–490. 16286016 10.1016/j.immuni.2005.09.015

[10] Li, Y. , Chen, S. , Chi, Y. , Yang, Y. , Chen, X. , Wang, H. , Lv, Z. et al., Kinetics of the accumulation of group 2 innate lymphoid cells in IL‐33‐induced and IL‐25‐induced murine models of asthma: a potential role for the chemokine CXCL16. Cell. Mol. Immunol. 2019 16: 75–86. 30467418 10.1038/s41423-018-0182-0 PMC6318283

[11] Schiering, C. , Krausgruber, T. , Chomka, A. , Frohlich, A. , Adelmann, K. , Wohlfert, E. A. , Pott, J. et al., The alarmin IL‐33 promotes regulatory T‐cell function in the intestine. Nature 2014 513: 564–568. 25043027 10.1038/nature13577 PMC4339042

[12] Bonilla, W. V. , Frohlich, A. , Senn, K. , Kallert, S. , Fernandez, M. , Johnson, S. , Kreutzfeldt, M. et al., The alarmin interleukin‐33 drives protective antiviral CD8(+) T cell responses. Science 2012 335: 984–989. 22323740 10.1126/science.1215418

[13] Baumann, C. , Bonilla, W. V. , Frohlich, A. , Helmstetter, C. , Peine, M. , Hegazy, A. N. , Pinschewer, D. D. et al., T‐bet‐ and STAT4‐dependent IL‐33 receptor expression directly promotes antiviral Th1 cell responses. Proc. Natl. Acad. Sci. USA 2015 112: 4056–4061. 25829541 10.1073/pnas.1418549112 PMC4386370

[14] Moussion, C. , Ortega, N. and Girard, J. P. , The IL‐1‐like cytokine IL‐33 is constitutively expressed in the nucleus of endothelial cells and epithelial cells in vivo: a novel ‘alarmin’? PLoS One 2008 3: e3331. 18836528 10.1371/journal.pone.0003331 PMC2556082

[15] Krishnamurty, A. T. and Turley, S. J. , Lymph node stromal cells: cartographers of the immune system. Nat. Immunol. 2020 21: 369–380. 32205888 10.1038/s41590-020-0635-3

[16] Nadafi, R. , Gago de Graca, C. , Keuning, E. D. , Koning, J. J. , de Kivit, S. , Konijn, T. , Henri, S. et al., Lymph node stromal cells generate antigen‐specific regulatory T cells and control autoreactive T and B cell responses. Cell Rep. 2020 30: 4110–4123. 32209472 10.1016/j.celrep.2020.03.007

[17] Chai, Q. , Onder, L. , Scandella, E. , Gil‐Cruz, C. , Perez‐Shibayama, C. , Cupovic, J. , Danuser, R. et al., Maturation of lymph node fibroblastic reticular cells from myofibroblastic precursors is critical for antiviral immunity. Immunity 2013 38: 1013–1024. 23623380 10.1016/j.immuni.2013.03.012 PMC7111182

[18] Onder, L. , Narang, P. , Scandella, E. , Chai, Q. , Iolyeva, M. , Hoorweg, K. , Halin, C. et al., IL‐7‐producing stromal cells are critical for lymph node remodeling. Blood 2012 120: 4675–4683. 22955921 10.1182/blood-2012-03-416859 PMC3952724

[19] Aparicio‐Domingo, P. , Cannelle, H. , Buechler, M. B. , Nguyen, S. , Kallert, S. M. , Favre, S. , Alouche, N. et al., Fibroblast‐derived IL‐33 is dispensable for lymph node homeostasis but critical for CD8 T‐cell responses to acute and chronic viral infection. Eur. J. Immunol. 2021. 10.1002/eji.201948413 32700362

[20] Welten, S. P. , Redeker, A. , Franken, K. L. , Oduro, J. D. , Ossendorp, F. , Cicin‐Sain, L. , Melief, C. J. et al., The viral context instructs the redundancy of costimulatory pathways in driving CD8(+) T cell expansion. Elife 2015 4: e07486. 10.7554/eLife.07486 PMC4558566 26263500

[21] Freeman, B. E. , Hammarlund, E. , Raué, H. P. and Slifka, M. K. , Regulation of innate CD8+ T‐cell activation mediated by cytokines. Proc. Natl. Acad. Sci. USA 2012 109: 9971–9976. 22665806 10.1073/pnas.1203543109 PMC3382521

[22] Simons, K. H. , de Vries, M. R. , Peters, H. A. B. , Jukema, J. W. , Quax, P. H. A. and Arens, R. , CD8+ T cells protect during vein graft disease development. Front. Cardiovasc. Med. 2019 6: 77. 31263704 10.3389/fcvm.2019.00077 PMC6584838

[23] Rodda, L. B. , Lu, E. , Bennett, M. L. , Sokol, C. L. , Wang, X. , Luther, S. A. , Barres, B. A. et al., Single‐cell RNA sequencing of lymph node stromal cells reveals niche‐associated heterogeneity. Immunity 2018 48: 1014–1028. 29752062 10.1016/j.immuni.2018.04.006 PMC5971117

[24] McLaren, J. E. , Clement, M. , Marsden, M. , Miners, K. L. , Llewellyn‐Lacey, S. , Grant, E. J. , Rubina, A. et al., IL‐33 augments virus‐specific memory T cell inflation and potentiates the efficacy of an attenuated cytomegalovirus‐based vaccine. J. Immunol. 2019 202: 943–955. 30635396 10.4049/jimmunol.1701757 PMC6341181

[25] Gao, X. , Wang, X. , Yang, Q. , Zhao, X. , Wen, W. , Li, G. , Lu, J. et al., Tumoral expression of IL‐33 inhibits tumor growth and modifies the tumor microenvironment through CD8+ T and NK cells. J. Immunol. 2015 194: 438–445. 25429071 10.4049/jimmunol.1401344 PMC4272901

